# Quantum Mechanics can be understood through stochastic optimization on spacetimes

**DOI:** 10.1038/s41598-019-56357-3

**Published:** 2019-12-27

**Authors:** Jussi Lindgren, Jukka Liukkonen

**Affiliations:** 1 Aalto University, Department of Mathematics and Systems Analysis, Espoo, Finland; 2 Nuclear and Radiation Safety Authority, STUK, Helsinki, Finland

**Keywords:** Applied mathematics, Quantum mechanics, Statistical physics

## Abstract

The main contribution of this paper is to explain where the imaginary structure comes from in quantum mechanics. It is shown how the demand of relativistic invariance is key and how the geometric structure of the spacetime together with the demand of linearity are fundamental in understanding the foundations of quantum mechanics. We derive the Stueckelberg covariant wave equation from first principles via a stochastic control scheme. From the Stueckelberg wave equation a Telegrapher’s equation is deduced, from which the classical relativistic and nonrelativistic equations of quantum mechanics can be derived in a straightforward manner. We therefore provide meaningful insight into quantum mechanics by deriving the concepts from a coordinate invariant stochastic optimization problem, instead of just stating postulates.

## Introduction

Since the inception of Quantum Mechanics (QM), there has been an on-going discussion on the ontology of the theory and its interpretations. In particular, there has been recently an intense debate on the validity of the so-called statistical interpretation of QM, see^1,2^. The ontological problem of QM is manifested especially clearly in the measurement problem. Therefore, understanding the physical meaning of the wave function is paramount. To understand the wave function, one needs to understand the equations of quantum physics.

The celebrated Schrödinger equation is mathematically close to the ordinary diffusion equation. What is the main difference is that time is imaginary, there is the Wick rotation. This means that classical and quantum are related partly by a rotation of 90 degrees in the complex plane (multiplying by the imaginary unit). The Schrödinger equation is given by:

1 $$i\hslash \frac{\partial \varphi ({\bf{x}},t)}{\partial t}=-\,\frac{{\hslash }^{2}}{2m}\Delta \varphi ({\bf{x}},t)+V({\bf{x}})\varphi ({\bf{x}},t)$$

For vectors we use the notation with bold fonts, so that for example **x** = (*x*, *y*, *z*). One of the key properties of the Schrödinger equation is that it is a linear, parabolic partial differential equation. Linearity is intimately related to the properties of de Broglie ‘matter’ waves. The Born rule then gives us the probability density as $\rho ({\bf{x}},t)=\varphi {\varphi }^{\ast }$, where the asterisk refers to complex conjugation.

The literature provides many heuristic ways to justify the equation, but most of the heuristics are not completely satisfactory in terms of understanding. The postulates of quantum mechanics are just stated, including and in particular the operator substitution rules for energy and momentum. Especially confusing is the imaginary nature of these differential operators. Even though the Schrödinger equation is adopted as a postulate of Quantum Mechanics in the literature, we argue that it can be derived in a meaningful manner and therefore from a didactical and pedagogical point of view, the postulate approach is not totally satisfying. The same challenges are omnipresent also in the sphere of relativistic quantum mechanics.

The stochastic optimal control approach to quantum mechanics can be traced back to Edward Nelson^3^. Among others, Yasue^4^ and Papiez^5^ have worked with stochastic control and quantum mechanics in the 1980s. Furthermore Rosenbrock and Ding have done quantum mechanics with control theory^6^. This study takes the control view as a starting point, where ultimately the Schrödinger equation is essentially the Hamilton-Jacobi-Bellman (HJB) equation from optimal control theory, when one takes into account relativistic coordinate-invariance of the action and demands linearity. Linearity of the Schrödinger equation is important due to the well-known properties of ‘matter’ waves, such as interference and superposition. The mathematical apparatus of linear operators yields also other useful tools such as spectral theory, eigenfunction expansions and so forth. In terms of more recent research, see the paper by Ohsumi^7^. Alas, what is missing also from Ohsumi’s paper is a proper and physically meaningful explanation why the Schrödinger equation is the diffusion equation in imaginary time. It should also be noted that recently it has been shown that the famous Heisenberg uncertainty relations seem to be inherent to stochastic systems in general, and they are not unique to quantum mechanical systems, see the recent paper^8^. There is also thread of literature, where stochastic quantization is incorporated with Special Theory of Relativity, see for example^9,10^, but analytic continuation is not explained, again.

We work in units so that we choose the reduced Planck’s constant to be $\hslash =1$. There are three space dimensions and one time dimension, i.e. we are working in the normal spacetime setting. Normal notation for contravariant and covariant tensors is used throughout, as well as the Einstein summation convention.

## Stochastic Classical Mechanics

We take classical mechanics as the starting point. In line with the existing literature, we assume that nature tries to minimize the classical action, so that

2 $$\mathop{{\rm{\min }}}\limits_{{\rm{paths}}}A={\int }_{t}^{T}\,(\frac{1}{2}m{v}^{2}-V({\bf{x}}))ds$$

over a finite time interval *t* ≤ *s* ≤ *T* and where the Lagrangian is the ordinary difference of kinetic and potential energies. Then one has the law of motion (Newton’s 2nd law) by inspecting the Euler-Lagrange equation. Instead, suppose that the path of the test particle obeys a Markov diffusion, so that

3 $$d{X}_{i}={v}_{i}ds+{\tilde{\sigma }}_{i}d{W}_{i}$$

where ${\tilde{\sigma }}_{i}$ is a scaling parameter and *W*_*i*_ is the standard, independent Wiener process, for all *i* = 1, 2, 3. This will make the above action integral a stochastic variable, and therefore one can postulate that nature tries to minimize the expected value (ensemble) for the action, this will lead to

4 $$\mathop{{\rm{\min }}}\limits_{{\rm{paths}}}S={E}_{tx}({\int }_{{\rm{t}}}^{T}\,(\frac{1}{2}m{v}^{2}-V(X))ds)$$

With the initial data *X*(*t*) = **x**, and where the velocity **v**(*s*, **x**(*s*)) is the Markov control policy. The expectation for the observable, which is the classical action, is understood as a conditional expectation, where we have some initial distribution and a transition probability measure. In essence, the transition probability is obtained from the Chapman-Kolmogorov equation:

5 $$p(y,s)=\int p(y,s|x,t)p(x,t)dx$$

Which means that the transition probability density is to be obtained as a sum over all paths, given the initial distribution *p*(*x*, *t*). Then for some observable *f*(*X*(*s*)) we have the expectation

6 $$E[f(X(s))]=\int f(y)p(y,s)dy$$

Which gives us the expected value for the observable again as a sum over all paths. There is an obvious link here with the path integral formulation of quantum mechanics. For good sources on the technical details of Markov processes and classical stochastic optimal control, see e.g.^11–13^. The key point is that the conditional expectation is an integral over space against a transition probability density. This is essential to understand when we require relativistic invariance from the action.

We therefore have a stochastic control problem which is linear-quadratic in control and it is straightforward to write down the corresponding HJB optimality PDE for the value function. The minimum value for the action with an optimal velocity path is denoted by *J*(**x**, *t*). The HJB equation can be shown to be

7 $$\frac{\partial J}{\partial t}-V({\bf{x}})-\frac{1}{2m}{(\nabla J)}^{2}+\frac{1}{2}{\sigma }^{2}\Delta J=0$$

with the optimal (Hamiltonian is minimized) velocity

8 $${\bf{v}}=-\frac{1}{m}(\nabla J)$$

or denoting the linear momentum by **p** we have

9 $${\bf{p}}=-\,(\nabla J)$$

and ${\sigma }^{2}={\tilde{\sigma }}_{1}^{2}+{\tilde{\sigma }}_{2}^{2}+{\tilde{\sigma }}_{3}^{2}$ and Δ is the ordinary Laplacian. The HJB equation and its derivations can be found from the excellent textbook^11^. It is worthwhile to note that the velocity is a gradient, where the potential is the value function of the HJB equation. As a curiosity, differentiating the HJB equation with respect to **x** gives the hydrodynamic representation, basically the time-reversed Navier-Stokes equation.

## Coordinate Invariance, Diffusions in the Minkowski Spacetime and Wick rotation

As we argue that relativistic invariance is one of the building blocks in understanding quantum physics, we need to consider diffusions not in just three spatial dimensions, but we need to allow the time variable to obey a diffusion process as well. This kind of reasoning makes sense: the time-dimension should be on an equal footing with the spatial dimensions. This kind of reasoning can be found for example in^14^. We therefore have the four-position describing events in the spacetime evolving according to the spatial diffusions as described above and additionally we have the temporal diffusion:

10 $$d(c{X}_{0})={u}_{0}ds+{\tilde{\sigma }}_{0}d{W}_{0}$$

where *s* is to be understood as the proper time, in line with special relativity. The factor *c*, i.e. speed of light, is needed also to balance the units, as in special relativity. We also have the spatial diffusions

11 $$d{X}_{i}={u}_{i}ds+{\tilde{\sigma }}_{i}d{W}_{i}$$

with *i* = 1, 2, 3. Therefore we have a 4-dimensional system that is to be controlled. The variable *X*_0_ represents time. A point or an event in the spacetime is represented by **x** = (*cx*_0_(*s*), *x*_1_(*s*), *x*_2_(*s*), *x*_3_(*s*)) in some reference frame so that events in the spacetime are parametrized by the proper time *s*.

Before we consider the HJB equation any further, we need to make sure that when performing stochastic optimal control on spacetimes, the expectation over spacetime is invariant with respect to coordinate transformations between reference frames. This demand means that the laws of physics are to be the same in any coordinate system, cf. general relativity and special theory of relativity. Given that there is an expectation operator, there is an integral over the spacetime with respect to a transition probability density (see the discussion above) and therefore one could consider the stochastic control problem as a classical field theory, where one integrates over spacetime. We can keep the multiple integral coordinate invariant by considering the general invariant scalar volume form:

12 $$dV=\sqrt{g}dc{x}_{0}d{x}_{1}d{x}_{2}d{x}_{3}$$

It is important to note that the volume form includes the effect of the metric tensor. The *g* inside the square root is the determinant of the metric tensor. We assume that we have the Minkowski spacetime, as it holds always locally on smooth Lorentzian manifolds, which is indeed relevant at Planck scales. The metric tensor can be represented by a diagonal matrix with entries

13 $${g}_{ij}=[\begin{array}{llll}-1 & 0 & 0 & 0\\ 0 & 1 & 0 & 0\\ 0 & 0 & 1 & 0\\ 0 & 0 & 0 & 1\end{array}]$$

The determinant *g* is clearly −1 and $\sqrt{g}=i$ and the line-element in the spacetime is:

14 $$d{r}^{2}={g}_{ij}d{X}^{i}d{X}^{j}=d{x}_{1}^{2}+d{x}_{2}^{2}+d{x}_{3}^{2}-{c}^{2}d{{\rm{x}}}_{0}^{2}$$

The line-element is a scalar as there are two contravariant vectors paired with the covariant metric tensor.

In order to form a relativistically invariant integral, we consider the four-velocity invariant as in^14^: ||*u*||^2^ = *g*_*ij*_*u*^*i*^*u*^*j*^. We then form the relativistically invariant integral as a direct extension of the classical mechanics Lagrangian in 4-dimensional spacetime (which is an expectation):

15 $$S=\mathop{\int }\limits_{{M}^{4}}\,{\int }_{\tau }^{T}\,(\frac{1}{2}m{g}_{ij}{u}^{i}{u}^{j}-V({\bf{x}}))dsp({\bf{x}},s)dV$$

with the invariant volume form: $dV=\sqrt{g}dc{x}_{0}d{x}_{1}d{x}_{2}d{x}_{3}$. Note that the value of the potential does not depend on the coordinate system chosen as it is an invariant scalar.

Therefore, the first key idea in this paper is that the Wick rotation or the analytic continuation to the imaginary axis in quantum mechanics and in the Schrödinger equation in particular comes from the invariant volume form on Lorentzian manifolds, as we have:

16 $$S=\mathop{\int }\limits_{{M}^{4}}\,{\int }_{\tau }^{T}\,(\frac{1}{2}m{g}_{ij}{u}^{i}{u}^{j}-V({\bf{x}}))dsp({\bf{x}},s)idc{x}_{0}d{x}_{1}d{x}_{2}d{x}_{3}$$

With the initial condition *X*(*τ*) = **x**. Therefore the inclusion of the Minkowskian metric volume form brings about the integrand which includes the imaginary unit. This is the essence how we can transform ourselves from the classical realm into the quantum world.

### The Hamiltonian and optimal feedback control

In this section we construct the Hamiltonian of the system. We follow the conventions as in^11^. The Hamiltonian function for the stochastic control problem is given by:

17 $$ {\mathcal H} =\mathop{{\rm{\sup }}}\limits_{{u}^{\mu }\in {\mathscr{U}}}(-\,{\nabla }_{\mu }J{u}^{\mu }- {\mathcal L} )$$

Where the Lagrangian is ${\mathcal L} =i(\frac{1}{2}m{g}_{\nu \mu }{u}^{\nu }{u}^{\mu }-V({\bf{x}}))$

The optimal control is the one that maximizes the Hamiltonian, so as a necessary condition, we demand:

18 $$\frac{\partial  {\mathcal H} }{\partial {u}^{\mu }}=0$$

Solving for the optimal feedback control, we have

19 $$-{\nabla }_{\mu }J-im{u}_{\mu }^{\ast }=0$$

Then

20 $${u}_{\mu }^{\ast }=-\,\frac{1}{im}{\nabla }_{\mu }J$$

Raising an index using the metric tensor, we obtain

21 $${u}^{\mu }=-\,\frac{1}{im}{\nabla }^{\mu }J$$

Substituting this optimal control back into the Hamiltonian, we get

22 $$ {\mathcal H} ({u}_{\mu }^{\ast })=\frac{1}{im}{\nabla }_{\mu }J{\nabla }^{\mu }J-\frac{1}{2}im(-\frac{1}{im}{\nabla }_{\mu }J)(-\frac{1}{im}{\nabla }^{\mu }J)+iV({\bf{x}})$$

23 $$ {\mathcal H} ({u}_{\mu }^{\ast })=\frac{1}{im}{\nabla }_{\mu }J{\nabla }^{\mu }J-\frac{1}{2}(\frac{1}{im}{\nabla }_{\mu }J{\nabla }^{\mu }J)+iV({\bf{x}})$$

24 $$ {\mathcal H} ({u}_{\mu }^{\ast })=\frac{1}{2im}{\nabla }_{\mu }J{\nabla }^{\mu }J+iV({\bf{x}})$$

The Hamiltonian is the kinetic energy (four-vectors instead of ordinary vectors) plus the potential energy, analytically continued due to the imaginary unit from the invariant volume form on Minkowski spacetime.

## Coordinate Invariance Together with Linearity Leads to Quantum Mechanics

We have specified a four-dimensional diffusion for the spacetime coordinates and now one needs to consider the respective Hamilton-Jacobi-Bellman equation. In principle it is straightforward, we follow^11,14^. In essence, we need to be careful with two things: first, we need to remember that the inner product is determined by the Minkowski metric so that covariant and contravariant objects are generally different, as the sign changes in the time-coordinate. Second, what used to be time, is now proper time. Additionally, we need to insert the imaginary unit properly into the HJB equation, due to the invariant volume form and thus due to the imaginary Hamiltonian and imaginary Lagrangian. Taking all this into account, the HJB equation is:

25 $$\frac{\partial J}{\partial \tau }-iV({\bf{x}})-\frac{1}{2im}{\nabla }_{\mu }J{\nabla }^{\mu }J+\frac{1}{2}{\sigma }^{2}{\nabla }_{\mu }{\nabla }^{\mu }J=0$$

Notice how in the equation we have both the contravariant and the covariant nabla operator and hence the Laplacian is the Laplace-Beltrami operator, although in this case as the metric tensor does not depend on the coordinates in the Minkowski spacetime, it is just the d’Alembertian. We have used Einstein summation convention and the index *μ* to represent the coordinates.

We can understand the equation better, when we notice that the nonlinear term is just representing the kinetic energy of the system via the four-momentum:

26 $$\frac{\partial J}{\partial \tau }-iV({\bf{x}})+iK+\frac{1}{2}{\sigma }^{2}{\nabla }_{\mu }{\nabla }^{\mu }J=0$$

or

27 $$\frac{\partial J}{\partial \tau }+i(K-V)+\frac{1}{2}{\sigma }^{2}{\nabla }_{\mu }{\nabla }^{\mu }J=0$$

where $K=\frac{{P}_{\mu }{P}^{\mu }}{2m}$ and *P*_*μ*_ = *i*▽_*μ*_*J* is four-momentum. The momentum comes from the optimal feedback control policy: ${u}_{\mu }=-\,\frac{1}{im}{\nabla }_{\mu }J\Leftarrow m{u}_{\mu }=i{\nabla }_{\mu }J$

Note how this definition for linear momentum explains the operator substitution postulate. The sign is reverted due to the fact that the HJB equation is solved backwards in time. Instead of postulating the operator substitution rules in a completely ad hoc manner, here we have derived them in a meaningful way.

We are looking for a linear PDE in general, as we are looking an equation describing ‘matter waves’, therefore we need to couple the scaling factor in such a way that the PDE

28 $$\frac{\partial J}{\partial \tau }-iV({\bf{x}})-\frac{1}{2im}{\nabla }_{\mu }J{\nabla }^{\mu }J+\frac{1}{2}{\sigma }^{2}{\nabla }_{\mu }{\nabla }^{\mu }J=0$$

becomes linear and we therefore choose:

29 $${\sigma }^{2}=\frac{i}{m}=-\frac{1}{im}$$

where the the variance term in the HJB equation is the following sum of the individual variances of the each diffusion component in the Minkowski spacetime (see the determination of *σ*^2^ in the book^11^):

30 $${\sigma }^{2}=-\,{\tilde{\sigma }}_{0}^{2}+{\tilde{\sigma }}_{1}^{2}+{\tilde{\sigma }}_{2}^{2}+{\tilde{\sigma }}_{3}^{2}$$

Note the minus sign stemming from the Minkowski metric. Then we must have

31 $${\sigma }^{2}=-\,{\tilde{\sigma }}_{0}^{2}+{\tilde{\sigma }}_{1}^{2}+{\tilde{\sigma }}_{2}^{2}+{\tilde{\sigma }}_{3}^{2}=\frac{i}{m}$$

Let

32 $${\tilde{\sigma }}_{1}^{2}+{\tilde{\sigma }}_{2}^{2}+{\tilde{\sigma }}_{3}^{2}=R\in {\mathbb{R}}$$

We can choose in particular ${\tilde{\sigma }}_{0}^{2}=R+\frac{1}{im}$ . Then this particular variance structure of the Minkowski spacetime implies that the variances are real in the spatial coordinates and that the diffusion scaling factor in the time coordinate is a proper complex number. This could be of further interest as such, because as ${\tilde{\sigma }}_{0}=\sqrt{R+\frac{1}{im}}$ is a proper complex number with real and imaginary parts, it implies that time can be understood as a two-dimensional object - it lives in the complex plane - and it has both a real component and a purely imaginary component. This can be seen from the temporal diffusion model:

33 $$d(c{X}_{0})={u}_{0}ds+{\tilde{\sigma }}_{0}d{W}_{0}={u}_{0}ds+\sqrt{R+\frac{1}{im}}d{W}_{0}$$

This ‘complex time’ is a mathematical consequence and requirement to linearise the HJB equation. The wave function is also complex-valued, but it is still a useful object in physics, whether or not it is ontologically ‘real’.

These considerations turn the HJB equation into

34 $$\frac{\partial J}{\partial \tau }-iV({\bf{x}})-\frac{1}{2im}({\nabla }_{\mu }J{\nabla }^{\mu }J+{\nabla }_{\mu }{\nabla }^{\mu }J)=0$$

Let us invoke a (Hopf-Cole) logarithmic transformation, so that $J=\,\log \,\varphi$ then the HJB equation becomes linear.

35 $$\frac{\frac{\partial \varphi }{\partial \tau }}{\varphi }-iV({\bf{x}})-\frac{1}{2im}(-\frac{1}{{c}^{2}}\frac{{\varphi }_{tt}}{\varphi }+\frac{{\varphi }_{xx}}{\varphi }+\frac{{\varphi }_{yy}}{\varphi }+\frac{{\varphi }_{zz}}{\varphi })=0$$

Finally, multiplying through with $i\varphi$, we obtain the following PDE:

36 $$i\frac{\partial \varphi ({\bf{x}})}{\partial \tau }=\frac{1}{2m}\square \varphi ({\bf{x}})-V({\bf{x}})\varphi ({\bf{x}})$$

where ☐ is the d’Alembertian partial differential operator. From this we can see that we have actually obtained the (time-reversed) Stueckelberg wave equation, which was invented already in 1941, see^15^. It can be understood as the Schrödinger equation in four dimensional Minkowski spacetime. Stueckelberg did not unfortunately explain either the imaginary structure of his generalised relativistic wave equation, he just postulated it. Stueckelberg’s wave equation is the foundation for what is called the approach of ‘Parameterized Relativistic Dynamics (PRD)’, see e.g.^16^.

The spacetime diffusion approach seems to be therefore connected also to considerations of antiparticles and particles^15^, where charge-reversal is related to time reversal. Nonlocality and the possible link with gravitation is considered for example in papers^17,18^ and references therein. In none of these papers, however, the current approach of coordinate invariant stochastic optimization is utilised as a teleological explanation for the resulting (complex) Stueckelberg field equations.

### The missing link between the Stueckelberg equation and the Dirac equation: the Telegrapher’s equation

In this section we derive the Telegrapher’s equation from the Stueckelberg equation above. Telegrapher’s equation is very important as it is a hyperbolic PDE from which Klein-Gordon and Dirac equations can be derived from, see^19^. We recall that in Special Relativity the proper time *τ* is defined as:

37 $$d\tau =\sqrt{1-\frac{{v}^{2}}{{c}^{2}}}dt$$

Therefore the Stueckelberg equation becomes

38 $$\frac{i}{\sqrt{1-\frac{{v}^{2}}{{c}^{2}}}}\frac{\partial \varphi ({\bf{x}})}{\partial t}=\frac{1}{2m}\square \varphi ({\bf{x}})-V({\bf{x}})\varphi ({\bf{x}})$$

Or in a more convenient form

39 $$\frac{1}{2m}\square \varphi ({\bf{x}})-\frac{i}{\sqrt{1-\frac{{v}^{2}}{{c}^{2}}}}\frac{\partial \varphi ({\bf{x}})}{\partial t}-V({\bf{x}})\varphi ({\bf{x}})=0$$

This shows that the HJB equation reduces to the Telegrapher’s equation in the present relativistic setting of optimal control. The Telegrapher’s equation is indeed appropriate, due to its property of finite speed of propagation, see e.g.^20^. This is required for a causal theory and therefore the present model of spacetime diffusion is superior to canonical stochastic control models in *R*^3^, as the respective HJB equation is in those contexts parabolic, thus leading to infinite speed of propagation. Moving into a relativistic optimal control setting hence represents somewhat a similar procedure as has been done in other physical contexts, such as in relativistic thermodynamics, see e.g.^21^.

The Klein-Gordon equation and the Dirac equation are closely related and even obtained from the Telegrapher’s equation, see the profound paper^19^. In this profound paper it is interesting that the authors also seem to struggle with the problem of the analytic continuation when they derive the Telegrapher’s equation using Poisson processes, see other approaches by^22,23^ and^24^. In the present paper no ad hoc analytic continuation is needed, as the imaginary unit comes naturally from the invariant volume form. It should be also noted that the Klein-Gordon equation is the stationary equation when one sets the partial derivative with respect to proper time to zero:

40 $$\square \varphi ({\bf{x}})-2mV({\bf{x}})\varphi ({\bf{x}})=0$$

Which is a hyperbolic PDE and manifestly Lorentz-covariant.

### Obtaining the Schrödinger equation in the nonrelativistic limit and the relationship between probability and energy

Consider the Telegrapher’s equation above:

41 $$\frac{1}{2m}\square \varphi ({\bf{x}})-\frac{i}{\sqrt{1-\frac{{v}^{2}}{{c}^{2}}}}\frac{\partial \varphi ({\bf{x}})}{\partial t}-V({\bf{x}})\varphi ({\bf{x}})=0$$

Passing to the nonrelativistic limit, *c*→∞, the proper time is just the ordinary time and the first term of the d’Alembertian goes to zero. This in turn gives us

42 $$i\frac{\partial \varphi ({\bf{x}})}{\partial t}=\frac{1}{2m}(\frac{{\partial }^{2}}{\partial {x}^{2}}+\frac{{\partial }^{2}}{\partial {y}^{2}}+\frac{{\partial }^{2}}{\partial {z}^{2}})\varphi ({\bf{x}})-V({\bf{x}})\varphi ({\bf{x}})$$

Which is the Schrödinger equation with time reversed, but from the time-symmetry properties of the Schrödinger equation we know that if $\varphi$ satisfies the above equation then its complex conjugate satisfies the canonical Schrödinger equation

43 $$i\frac{\partial {\varphi }^{\ast }({\bf{x}})}{\partial t}=-\frac{1}{2m}\Delta {\varphi }^{\ast }({\bf{x}})+V({\bf{x}}){\varphi }^{\ast }$$

Finally, it is worth considering that there is an interesting natural link between the Born rule and the minimal expected action, because we have the complex algebraic identity ${J}^{\ast }=\,\log \,{\varphi }^{\ast }$, from which it immediately follows that the Born rule gives $p=\varphi {\varphi }^{\ast }={e}^{2a}$ where we assume that the value function is of the form *J* = *a*(*x*, *y*, *z*, *t*) + *ib*(*x*, *y*, *z*, *t*).

## Conclusion and Discussion

This study shows that the imaginary nature of various variables in quantum mechanics is due to the structure of the Minkowski metric. This paper derives the Stueckelberg relativistic wave equation and analytically continued Telegrapher’s equation directly from a stochastic optimal control scheme, where the four-position evolves in a random way. The equations are obtained as a transformed solution of the HJB equation, when one demands coordinate invariance and couples the amplitude of the noise to the mass of the particle in such a way that the logarithmic transformation gives a linear HJB equation. In terms of future research, perhaps one should try to establish the wave equation in a general curved spacetime and thus generalise the metric into a more general form. The method of choice could be stochastic geometric control. This could be a way to combine general relativity and quantum mechanics, perhaps.

In line with^1^, the results presented in this paper do not therefore support the interpretative thesis given by the PBR theorem, which claims to rule out the statistical interpretation of the quantum state^2^. Thus, we advocate for a realistic interpretation of quantum mechanics. The model presented in this paper suggests that the test particle is moving under the influence of an external random spacetime force. This random movement of the particle induces the transition probability distribution. This means that quantum mechanics can be understood as a statistical theory. In literature there are some conjectures what could be the reason for this random force. See for example the profound paper^25^ as well as^26^. Therefore, one could make the conjecture that quantum mechanics or quantum field theory is only a phenomenological theory and the reason for the statistical nature lies within the stochastic nature of the spacetime itself^27^. If the spacetime and its metric is stochastic at Planck scales, it could produce the illusion of random movement, which could be phenomenologically modelled with stochastic differential equations in the spacetime. In line with General Relativity, this could mean in essence that the energy sources in the space-time have a random character ie. the stress-energy tensor has a random character, see^28^, which could come from various disturbances, such as vacuum or zero field radiation. Future research avenues in this regard should include at least random metrics, i.e. metric tensors represented by random matrices.

As elaborated in^27^, the origin of the Born rule has been somewhat ambiguous till today. According to the stochastic control paradigm presented in this paper, the Born rule for the test particle is related naturally to real part of the minimal expected action. This connection between probability, the optimal action and thus the wave function can be understood by noting that the optimal diffusion drift velocity depends on the negative gradient of the value function. The spacetime diffusion process takes the route which minimizes the expected action; this is the essence of how (transition) probability is tied to energy minimization. We firmly base our beliefs on the realistic philosophy of quantum mechanics, where reality exists independently of the observer. This inclination is put forward especially lucidly by Sir Karl Popper^29^.
